# Population genetic structure, genetic diversity, and natural history of the South American species of *Nothofagus* subgenus *Lophozonia* (Nothofagaceae) inferred from nuclear microsatellite data

**DOI:** 10.1002/ece3.1108

**Published:** 2014-05-19

**Authors:** Rodrigo Vergara, Matthew A Gitzendanner, Douglas E Soltis, Pamela S Soltis

**Affiliations:** 1 Department of Biology, University of FloridaGainesville, Florida, 32611; 2 Florida Museum of Natural History, University of FloridaGainesville, Florida, 32611

**Keywords:** Chile, *Nothofagus alpina*, *Nothofagus glauca*, *Nothofagus nervosa*, *Nothofagus obliqua*, SSR

## Abstract

The effect of glaciation on the levels and patterns of genetic variation has been well studied in the Northern Hemisphere. However, although glaciation has undoubtedly shaped the genetic structure of plants in the Southern Hemisphere, fewer studies have characterized the effect, and almost none of them using microsatellites. Particularly, complex patterns of genetic structure might be expected in areas such as the Andes, where both latitudinal and altitudinal glacial advance and retreat have molded modern plant communities. We therefore studied the population genetics of three closely related, hybridizing species of *Nothofagus* (*N*. *obliqua*, *N. alpina*, and *N. glauca*, all of subgenus *Lophozonia*; Nothofagaceae) from Chile. To estimate population genetic parameters and infer the influence of the last ice age on the spatial and genetic distribution of these species, we examined and analyzed genetic variability at seven polymorphic microsatellite DNA loci in 640 individuals from 40 populations covering most of the ranges of these species in Chile. Populations showed no significant inbreeding and exhibited relatively high levels of genetic diversity (*H*_E_ = 0.502–0.662) and slight, but significant, genetic structure (*R*_ST_ = 8.7–16.0%). However, in *N. obliqua*, the small amount of genetic structure was spatially organized into three well-defined latitudinal groups. Our data may also suggest some introgression of *N. alpina* genes into *N. obliqua* in the northern populations. These results allowed us to reconstruct the influence of the last ice age on the genetic structure of these species, suggesting several centers of genetic diversity for *N. obliqua* and *N. alpina*, in agreement with the multiple refugia hypothesis.

## Introduction

The current genetic structure and diversity of natural plant populations can be seen as the resulting product of the interaction between biology, geography, and climatic change (Hewitt 2000). Important biological factors include the breeding system, life form, seed dispersal, and pollination mechanism of the species (Hamrick 1982; Hamrick and Godt 1996), which together with the geographical range influence the level of isolation by distance (Wright 1943) and the effectiveness of geographical barriers against colonization and pollen flow. In addition, the dramatic climatic changes that occurred during the ice ages in the Quaternary had major effects on the patterns of genetic diversity not only in the boreal and temperate regions of the Northern Hemisphere (Soltis et al. 1997; Hewitt 2000), but also in austral and temperate regions in the Southern Hemisphere (Ogden 1989; Premoli et al. 2003; Marchelli and Gallo 2004; Azpilicueta et al. 2009; Worth et al. 2009; Mathiasen and Premoli 2010).

The west coast of southern South America in Chile and western Argentina between 33°00′S and 41°30′S is crossed longitudinally by two mountain ranges separated by the Central Valley. The Coastal Range has altitudes between 500 and 2000 m above sea level (m a.s.l.), and the Andes have altitudes between 3000 and 6000 m a.s.l., both becoming generally lower from north to south. The Mediterranean forests are found between 33°00′S and 36°30′S, and the temperate rainforests south of 37°30′S, with an ecotonal zone – the transitional forests – in between (Donoso 1982; Veblen and Schlegel 1982).

**Figure 1:**
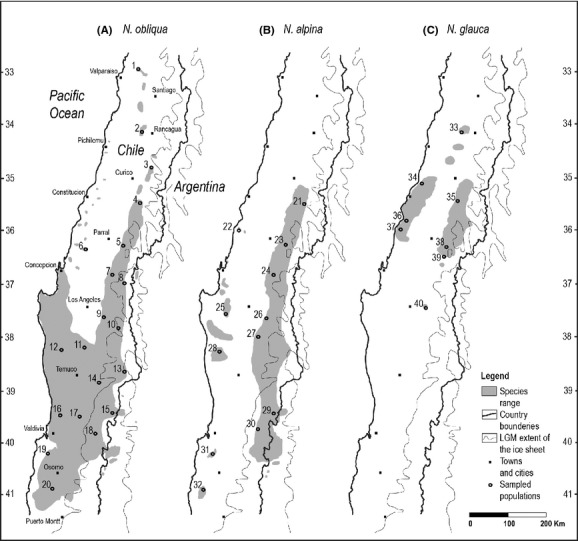
Range of distribution for (A) *Nothofagus obliqua*, (B) *N. alpina*, and (C) *N. glauca* in gray, showing the location of the sampled populations used in our study. Last glacial maximum extent of the ice sheet obtained from Hollin and Schilling (1981).

*Nothofagus obliqua* (Mirb.) Oerst., *N. alpina* (Poepp. et Endl.) Oerst. (= *N. nervosa*), and *N. glauca* (Phil.) Krasser. are sympatric South American endemics. Together with two species from Australia (*N. cunninghamii* (Hook.) Oerst. and *N. moorei* (Muell.) Krasser.) and one from New Zealand (*N. menziessii* (Hook.) Oerst.), these species constitute subgenus *Lophozonia* (Manos 1997). This group of deciduous species grows in both Mediterranean and temperate rainforest regions in Chile and adjacent areas in Argentina, occupying different elevations from the Central Valley to the Coastal and Andean mountain ranges (Ormazabal and Benoit 1987). Of the three species, *N. obliqua* has the most extensive geographical distribution, covering nearly 1000 km in longitude, *N. alpina* has an intermediate range of 700 km, and *N. glauca* has a narrower distribution covering approximately 400 km (Fig. 1).

These three species are tall, long-lived trees easily reaching 30 m in height and 300 years of age. These monoecious species have anemophilous pollination and a largely outcrossing breeding system (Riveros et al. 1995; Gallo et al. 1997; Ipinza and Espejo 2000). An important difference in reproductive biology among the three species is seed dispersal, which is predominantly accomplished by gravity in *N. glauca* and by a combination of wind and gravity in *N. obliqua* and *N. alpina* (Donoso 1993).

The current habitat of these species was largely affected by repeated glaciations during the Quaternary, influencing the distribution of forests. During the last glacial maximum (LGM ≈ 20,000 year ago), glaciers covered a large proportion of the current forest, and additionally, palynological research has identified periglacial effects (Villagran et al. 1995).

The Central Valley of central Chile (33°00′–36°00′S) is characterized by warm and dry summers and supports sclerophyllous forests. However, with more precipitation at higher elevations, it is possible to find populations of *Nothofagus* spp. that form presumably relict forests in the northern limit of their distributions, such as the *N. obliqua* island forests in the highlands of the Coastal Range between 33°00′ and 34°00′S (Donoso 1993; Villagran 2001). During the LGM, glaciers reached altitudes as low as 1200–3000 m lower than today, creating climatic conditions that favored the colonization of valleys by *Nothofagus* (Heusser 1990) and probably eradicated *Nothofagus* from the mountains. The Central Valley then could have served as one large panmictic glacial refugium or perhaps as several isolated refugia. The end of the ice age (10,000 years ago) gradually brought drier and warmer conditions to the area, pushing *Nothofagus* forests to their current distributions in the mountains.

Similar events occurred in the middle portion of the ranges of the three species (36°00′–39°00′S). Today, isolated populations of *N. alpina*, which has its main distribution in the Andes, occur on the top of the Nahuelbuta Mountains in the Coastal Range. These forest islands have been interpreted as the remnants of glacial populations that were once growing in the Central Valley during the ice age (Villagran 2001). Unlike central Chile, this area is currently wet enough for some other *Nothofagus* species (e.g., *N. obliqua*) to live in the Central Valley. According to pollen records, recolonization of Nahuelbuta by *Nothofagus* spp. started about 6000 years ago in the Holocene (Villagran 2001).

Finally, the area south of 40°00′S experienced different periglacial effects. The proximity of the glaciers during the last ice age allowed only the most cold-resistant and hygrophilous forest elements to survive in discontinuous populations in lowland sites in the Central Valley and in the Coastal Range (Villagran 2001). Vegetation was dominated by nonarboreal taxa mixed with these cold-resistant and hygrophilous species resembling a parkland with varying degrees of openness (Villagran et al. 1995; Moreno et al. 1999). Gradually, after 14,200 years ago, more mesic taxa started arriving in the area. Thermophyllous forest taxa (e.g., *N. obliqua*, *N. alpina*) might have expanded slowly to this area in the Holocene (after 10,000 years ago) when climatic conditions started to be warm and moist enough to support that vegetation (Moreno et al. 1999), which suggests that the refugia for those taxa were probably localized north of 40°00′S, in the Coastal Range and the Central Valley.

The hypothesis of glacial refugia for *N. obliqua* and *N. alpina* localized north of 40°00′S in places including the valleys near Nahuelbuta (Villagran 2001) or Rucañancu (39°30′S) in the Andes piedmont (Villagran 1991) is supported by the extant pollen records. However, there is the possibility that these species survived in low numbers in multiple scattered refugia associated with favorable microclimates at higher altitudes and latitudes, without leaving any trace in the pollen record (Markgraf et al. 1996). This latter hypothesis is supported directly by genetic studies in *N. obliqua* (Azpilicueta et al. 2009) and *N. alpina* (Marchelli et al. 1998; Marchelli and Gallo 2006; Carrasco et al. 2009) and indirectly by genetic studies in other tree species in the area (Allnutt et al. 1999; Premoli et al. 2000, 2003; Bekessy et al. 2002; Nunez-Avila and Armesto 2006) and southward (Pastorino et al. 2009; Mathiasen and Premoli 2010).

In accordance with *Nothofagus* anemophilous pollination, genecological studies on *N. obliqua* (Donoso 1979a) and *N. alpina* (Donoso 1987) and population genetics studies conducted using nuclear markers in *N. alpina* (Pineda 2000; Carrasco and Eaton 2002; Carrasco et al. 2009) show a north-to-south clinal pattern of variation produced by large-scale pollen flow and a climatic cline between the northern and southern populations. This pollen flow also facilitates interbreeding among species, generating natural hybrids in specific environmental conditions (i.e., *N. alpina* × *N. obliqua* (Donoso et al. 1990; Gallo et al. 1997; Marchelli and Gallo 2001) and *N. obliqua* x *N. glauca* (= *N. leonii* Espinosa) (Donoso 1979b)). Thus, anywhere these species grow in close proximity, there is a potential for hybridization and introgression among them. Likewise, this extensive pollen flow has been shown to maintain relatively high levels of genetic variability and low population differentiation in *N. alpina* and in most *Nothofagus* species (Table 1).

**Table 1:** Genetic variability and differentiation assessed with nuclear genetic markers in other similar studies

Species	Range	Marker	*K*	*N*	Loci	*A*	*H*_E_	*F*_ST_	Reference
From genus *Nothofagus*									
*N. alpina* (= *N. nervosa*)	Reg	Allozymes	22	19	7	2.9	0.484	9.6	Pineda 2000
		Allozymes	18	36	10	3.0	0.289	5.1	Carrasco and Eaton 2002
		RAPDs	22	27	33	–	0.150	12.4	Carrasco et al. 2009
		Allozymes	11	112	8	2.3	0.173	3.8	Marchelli and Gallo 2001
		Allozymes	20	115	8	3.4	0.180	5.2	Marchelli and Gallo 2004
		Allozymes	2	71	6	1.9	0.126	–	Milleron et al. 2008
		Allozymes	2	30	6	1.6	0.163	–	Milleron et al. 2008
		Microsats	2	71	3	4.3	0.474	–	Milleron et al. 2008
		Microsats	2	30	3	3.8	0.474	–	Milleron et al. 2008
		Microsats	14	35	7	4.4	0.452	6.1	Azpilicueta et al. 2013
*N. obliqua*	Reg	Microsats	10	34	7	4.3	0.455	4.9	Azpilicueta et al. 2013
		Allozymes	14	143	7	2.2	0.223	5.1	Azpilicueta and Gallo 2009
*N. alessandrii*	Narr	Allozymes	7	27	7	1.8	0.182	25.7	Torres-Diaz et al. 2007
*N. nitida*	Narr	Allozymes	4	42	15	1.3	0.045	4.7	Premoli 1997
*N. betuloides*	Reg	Allozymes	4	28	15	1.5	0.116	12.0	Premoli 1997
*N. dombeyi*	Reg	Allozymes	5	34	15	1.6	0.093	7.4	Premoli 1997
*N. pumilio*	Reg	Allozymes	41	29	7	1.4	0.070	20.0	Mathiasen and Premoli 2010
		Allozymes	6	90	5	2.0	0.084	–	Mathiasen and Premoli 2013
		Microsats	6	50	5	3.7	0.496	–	Mathiasen and Premoli 2013
*N. antarctica*	Reg	Allozymes	12	48	2	2.7	0.185	11.0	Pastorino et al. 2009
		Allozymes	28	36	8	2.2	0.207	18.8	Acosta et al. 2012
*N. truncata*	Reg	Allozymes	30	57	5	1.3	0.051	4.9	Haase 1992
*N. menziesii*	Reg	Allozymes	5	52	15	1.5	0.116	–	Haase 1993
*N. moorei*	Narr	ISSRs	20	7	42	1.8	0.168	10.4	Taylor et al. 2005
From other related genera									
*Fagus sylvatica*	Wide	Microsats	10	130	4	14.9	0.829	5.8	Buiteveld et al. 2007
*F. japonica*	Reg	Microsats	16	34	13	8.6	0.659	2.3	Hiraoka and Tomaru 2009
*Quercus glauca*	Wide	Microsats	10	19	4	6.5	0.741	4.2	Lee et al. 2006
*Q. macrocarpa*	Wide	Microsats	14	34	5	11.2	0.864	2.7	Craft and Ashley 2007
*Q. petraea*	Wide	Microsats	5	60	6	8.6	0.755	20.1	Bruschi et al. 2003
		Microsats	7	30	13	7.0	0.797	0.8	Muir et al. 2004
*Q. semiserrata*	Wide	Microsats	10	39	8	8.2	0.679	12.0	Pakkad et al. 2008
*Q. garryana*	Reg	Microsats	22	15	7	4.9	0.597	4.9	Marsico et al. 2009

Range (Hamrick and Godt 1996): wide, widespread; reg, regional; narr, narrow; *K*, number of populations; *N*, average sample size per population; loci, number of loci effectively analyzed; *A*, mean number of alleles per locus; *H*_E_, average expected heterozygosity; *F*_ST_, Wright's *F*_ST_ (Wright 1965) or its equivalent *G*_ST_ (Nei 1973) in percentage.

Our goal is to evaluate, through the use of nuclear microsatellite markers, the levels of genetic diversity and structure of the three South American species of subgenus *Lophozonia* (*Nothofagus*) to infer how these genetic parameters are influenced by climatic change, geography, and biological factors. Thus, in this study we will compare results obtained here with studies using other biparentally inherited markers in *N. alpina*: allozymes (Pineda 2000; Carrasco and Eaton 2002) and RAPDs (Carrasco et al. 2009). Furthermore, our microsatellite data will also complement recent studies on *N. obliqua* (Azpilicueta et al. 2009) and *N. alpina* (Marchelli and Gallo 2006) based on chloroplast DNA, which is maternally inherited and traces exclusively colonization by seed dispersal. In contrast, microsatellite markers can detect both pollen flow and seed dispersal and therefore allow for fine-scale analysis of local and regional patterns of genetic diversity (Selkoe and Toonen 2006).

We hypothesize that populations of the three species growing in the Mediterranean forests between 33°00′S and 36°30′S may have reduced genetic diversity and higher levels of differentiation due to their proposed isolation since the end of the last glacial period (Heusser 1990; Villagran 2001). We further expect to see lower genetic diversity in the narrowly distributed *N. glauca* than in the more widespread *N. obliqua* and *N. alpina*. Finally, we do not expect to see a north-to-south pattern of diminishing variation due to founder effects in the colonization after the LGM as in the patterns often seen in plants in the Northern Hemisphere (Soltis et al. 1997, 2006; Taberlet et al. 1998; Hewitt 2000; Petit et al. 2003). Instead, we predict different centers of variation along the current distribution of the species in line with the multiple refugia hypothesis for South America (Markgraf et al. 1996; Premoli et al. 2000).

## Materials and Methods

### Study sites, sample collection, and storage

We sampled populations across most of the distributional range of each species in Chile, including the latitudinal and altitudinal variation observed for each species (Fig. 1). We collected samples of fresh tissue from 16 individuals in each of 20 populations of *Nothofagus obliqua*, 12 populations of *N. alpina*, and 8 populations of *N. glauca* (Table 2). In all cases, we only collected samples from pure individuals, that is, individuals with an unambiguous morphological identity for each species. We included two newly described populations that were outside the known range of distribution before 2001, one for *N. alpina* (Sepulveda and Stoll 2003) and one for *N. glauca* (Le-Quesne and Sandoval 2001), and regarded the populations described as *N. macrocarpa* by Vazquez and Rodriguez (1999) (Table 2) as *N. obliqua*, following Donoso (1979a). We did not sample populations from Argentina.

**Table 2:** List of sampled populations for *Nothofagus obliqua*, *N. alpina*, and *N. glauca*

#	Population	Location	Latitude (S)^1^	Longitude (W)^1^	Elevation (m a.s.l.)^1^, ^2^	Source of plant material	Tissue collected
*N. obliqua*							
1	La Campana^3^	Coast	32°58′	71°07′	1380	Natural population	Buds
2	Loncha^3^	Coast	34°09′	70°57′	870	Natural population	Buds
3	Bellavista^3^	Andes	34°46′	70°44′	950	Natural population	Buds
4	Siete Tazas Alto^3^	Andes	35°28′	70°59′	1460	Natural population	Buds
5	Bullileo Alto	Andes	36°20′	71°23′	1150	Natural population	Buds
6	Ninhue	Coast	36°23′	72°25′	150	Provenance trial	Leaves
7	Recinto	Andes	36°51′	71°37′	710	Provenance trial	Leaves
8	Reserva Ñuble	Andes	37°06′	71°15′	1890	Provenance trial	Leaves
9	Santa Barbara	Andes	37°40′	71°59′	430	Provenance trial	Leaves
10	Ralco	Andes	37°51′	71°33′	810	Provenance trial	Leaves
11	Victoria	Central Valley	38°14′	72°18′	480	Provenance trial	Leaves
12	Pichipillahuen	Coast	38°17′	73°04′	450	Provenance trial	Leaves
13	Galletue	Andes	38°40′	71°18′	1450	Provenance trial	Leaves
14	Cunco	Andes	38°52′	72°00′	410	Provenance trial	Leaves
15	Curarrehue	Andes	39°25′	71°35′	910	Provenance trial	Leaves
16	Cruces	Coast	39°32′	73°05′	160	Provenance trial	Leaves
17	Malalhue	Central Valley	39°33′	72°33′	140	Provenance trial	Leaves
18	Choshuenco	Andes	39°50′	72°05′	220	Provenance trial	Leaves
19	Llancacura	Coast	40°16′	73°18′	60	Provenance trial	Leaves
20	Purranque	Coast	40°55′	73°11′	90	Provenance trial	Leaves
*N. alpina*							
21	Siete Tazas	Andes	35°29′	70°53′	760	Provenance trial	Leaves
22	Tregualemu	Coast	36°03′	72°40′	510	Natural population	Buds
23	Bullileo Alto	Andes	36°18′	71°24′	1650	Provenance trial	Leaves
24	Recinto	Andes	36°50′	71°36′	810	Provenance trial	Leaves
25	Nahuelbuta	Coast	37°31′	72°52′	940	Provenance trial	Leaves
26	Santa Barbara	Andes	37°44′	71°54′	430	Provenance trial	Leaves
27	Jauja	Andes	38°02′	72°02′	670	Provenance trial	Leaves
28	Pichipillahuen	Coast	38°18′	73°03′	420	Provenance trial	Leaves
29	Curarrehue	Andes	39°26′	71°35′	840	Provenance trial	Leaves
30	Releco	Andes	39°51′	71°55′	1160	Provenance trial	Leaves
31	Llancacura	Coast	40°17′	73°21′	380	Provenance trial	Leaves
32	Hueyusca	Coast	40°59′	73°30′	410	Provenance trial	Leaves
*N. glauca*							
33	Loncha	Coast	34°10′	71°01′	1110	Natural population	Buds
34	Alto Huelon	Coast	35°06′	72°04′	200	Natural population	Buds
35	Siete Tazas	Andes	35°26′	71°03′	830	Natural population	Buds
36	Los Ruiles	Coast	35°50′	72°30′	220	Natural population	Buds
37	Tregualemu	Coast	35°59′	72°40′	510	Natural population	Buds
38	Bullileo	Andes	36°18′	71°24′	710	Natural population	Buds
39	Alico	Andes	36°35′	71°28′	620	Natural population	Buds
40	Quilleco	Andes	37°28′	71°58′	340	Natural population	Buds

1 The coordinates and elevation of the source populations sampled for the provenance trials are only a rough approximation. There are no records of the exact position of the mother trees harvested to plant the trials.

2 Elevations were obtained entering geographic coordinates in a digital elevation model based on Shuttle Radar Topography Mission (SRTM) Finished 3 arc-second (90 m) raster elevation dataset.

3 Populations described as *N. macrocarpa* by Vazquez and Rodriguez (1999).

For *N. obliqua* and *N. alpina*, we obtained samples mostly from progeny-provenance trials, where we collected approximately 5 g of fresh leaf tissue from each tree in January 2004. These trials were established using known seed sources in the spring of 2000 by the FONDEF D96/1052 UACH-INFOR project in Fundo Arquilhue, Valdivia province, Los Ríos Region, Chile (40°14′S, 72°03′W, 304 m a.s.l.), consisting of 31 and 14 *N. obliqua* and *N. alpina* populations, respectively. We sampled populations where there were typically 10 mother trees (i.e., open-pollinated families) represented by five planted individuals per family; therefore, at each population, we sampled one offspring for each mother tree, and the remaining six sampled individuals are half-siblings to one of the originally sampled individuals. For *N. glauca*, and some populations of *N. obliqua* and *N. alpina*, we collected dormant buds in July 2003 from randomly selected trees separated, when possible, by at least 20 meters in natural populations (Table 2). All samples were dried and stored in silica gel and transported to the Laboratory of Molecular Systematics and Evolutionary Genetics at the Florida Museum of Natural History, University of Florida, USA, for DNA extraction and genotyping.

### DNA extraction

After pulverizing buds and leaf tissue in a bead mill, we extracted total genomic DNA using a modified CTAB protocol for silica-dried tissue (Doyle and Doyle 1987). We resuspended the DNA pellets in 100 *μ*L TE buffer, quantified DNA concentration using a NanoDrop® ND-1000 Spectrophotometer (Thermo Fisher Scientific, Inc., Waltham, MA), diluted samples to the concentrations of 50–200 ng/*μ*L, and stored samples at −20°C until use.

### PCR amplifications and genotyping

After DNA extraction, we used microsatellite markers to assess genetic variability. We screened 22 microsatellite loci: 14 loci developed for *N. cunninghamii* (Jones et al. 2004), three developed for *N. glauca* or *N. obliqua* (Azpilicueta et al. 2004), and five for *N. alpina* (Marchelli et al. 2008). Polymerase chain reactions (PCR) followed Jones et al. (2004), with a labeled M13-tail primer, using 384-well plates and a Bio-Rad Thermo Cycler (Bio-Rad Laboratories, Inc., Hercules, CA). After preliminary amplifications, we selected seven loci that were polymorphic and amplified consistently across species and populations at a standardized annealing temperature of 52°C and 2.0 mmol/L MgCl_2_ (Table 3).

**Table 3:** Microsatellite loci analyzed in *Nothofagus obliqua*, *N. alpina*, and *N. glauca*

				Total alleles per locus		
				
Locus	Primer sequences (5′–3′)	Repeat of reported allele	Size^1^	*N. obliqua*	*N. alpina*	*N. glauca*
*ncutas04*^2^	F: CTCCCGTGAGAAGGTTTGAAT	(CA)_11_	337–357	1	1	10
	R: AATGGGCATATGGTTATTGTGATAG					
*ncutas06*^2^	F: TTTCCCTCCATGAATACTTG	(CT)_14_	357–409	27	9	8
	R: AATGGCTTGATATTGTTACC					
*ncutas08*^2^	F: TTGAATGGCTTGACTTGTAA	(AC)_12_	217–233	7	7	5
	R: GATGGGTGAGAATTTTGACT					
*ncutas12*^2^	F: GCATCATCCCATCCTAAGTTAT	(CA)_16_	216–250	19	9	6
	R: CTGAACACTGGCATCTTTAATG					
*ncutas13*^2^	F: TAACCCACCACTCTTGCCGAAGT	(CT)_16_	304–358	21	25	12
	R: GGAACGGCCTCCACATCTCA					
*ncutas22*^2^	F: GATGGGGTTATCATAGGTGTCGT	(CT)_14_(AC)_11_	292–326	18	11	14
	R: TCAGCGAGAATTCCTTTGATGTA					
*NnBIO111*^3^	F: TATGTGAACGCGTCTGCTTC	(GT)_2_A(GT)_10_	134–162	–	10	5
	R: CGCTCTTCAGACCAGAAAGG					

1 Size range in our study (bp).

2 Developed by Jones et al. (2004).

3 Developed by Marchelli et al. (2008).

We genotyped the samples for all selected loci using an ABI 3730xl DNA Analyzer (Applied Biosystems, Inc., Foster City, CA) at the ICBR facility, University of Florida. We used four fluorescent dyes to label the M13 primer to combine four loci in each run by pooling them together in each well of a 96-well plate. We performed fragment analysis and scoring of alleles using GeneMapper® 4.0 (Applied Biosystems, Inc.), repeating unsuccessful amplifications once and treating second-round failures as missing data.

### Data analysis

#### Detection of scoring errors

Three types of errors – stuttering, large-allele dropouts, and null alleles – are common in microsatellite data and can create scoring bias (Dewoody et al. 2006). We attempted to mitigate the effects of scoring errors by identifying and correcting them using Micro-Checker 2.2.3 (van Oosterhout et al. 2004). We analyzed each locus/population combination using the Bonferroni (Dunn-Sidak)-adjusted 95% confidence interval in the Monte Carlo simulations to detect deviations from expected allele distributions. In the cases in which there was evidence of null alleles, we adjusted the allele and genotype frequencies following the procedure suggested in Micro-Checker. We used this adjusted dataset for all further analyses.

#### Linkage disequilibrium analysis

We conducted our population genetics analyses assuming that microsatellite loci are randomly distributed and independent of each other. We employed GenePop 4.0 (Rousset 2008) to perform a linkage disequilibrium (LD) analysis using the log-likelihood ratio statistic (*G*-test) and a Markov chain algorithm with 20 batches and 5000 iterations per batch on each population to detect linkage between pairs of loci. For comparison, we used the likelihood ratio LD procedure (with 1000 permutations) in Arlequin 3.11 (Excoffier et al. 2005) and applied the standard Bonferroni technique to obtain the proper significance for multiple comparisons (Rice 1989).

#### Genetic diversity within populations

For each species, we calculated intrapopulational diversity indices using Arlequin 3.11 (Excoffier et al. 2005) and GenePop 4.0 (Rousset 2008), including the number of alleles per locus (*A*), observed (*H*_O_) and expected (*H*_E_) heterozygosity, inbreeding coefficient (*F*_IS_), and allele size-based inbreeding coefficient (*ρ*_IS_). The parameter *A* is equivalent to allelic richness when, as in this study, sample sizes among populations are similar, and it is the most important parameter when evaluating glacial refugia (Widmer and Lexer 2001). We looked for deviations from Hardy–Weinberg equilibrium (HWE) with HWE exact tests, using the Markov chain (MC) algorithm (chain length: 1,000,000, dememorization steps: 100,000) in Arlequin 3.11 (Excoffier et al. 2005), and applied the standard Bonferroni technique (Rice 1989) to correct for multiple loci. We also tested for evidence of recent genetic bottlenecks using Bottleneck 1.2 (Cornuet and Luikart 1996) assuming the stepwise mutation model (SMM), running 1000 replications, and based on a sign test. We used SAS software 9.2 (SAS 2008) to test for differences in diversity indices (i.e., *A* and *H*_E_) across latitude and elevation obtaining Pearson correlation coefficients (*r*), and among species, locations (i.e., Coast, Andes), latitudinal groups (i.e., North, South), glacial refugia hypothesis (i.e., 37°30′S to 40°00′S, south of 40°00′S), and sources of plant material (i.e., natural population, provenance trial), using nonparametric Kruskal–Wallis tests. Additionally, we tested for differences in inbreeding coefficients (*F*_IS_ and *ρ*_IS_) between sources of plant material. We applied the standard Bonferroni technique (Rice 1989) in all of these analyses.

#### Genetic structure

To investigate the patterns of within-species genetic structure, we first carried out an individual-based approach assuming no specific mutation model using Bayesian clustering in the program Structure 2.3.2 (Pritchard et al. 2000). Following Falush et al. (2003), we used the admixture model with correlated allele frequencies. We analyzed the three species separately with 320, 192, and 128 individuals and 20, 12, and 8 sampled populations for *N. obliqua*, *N. alpina*, and *N. glauca*, respectively, without including population information in the analyses. We ran Structure at multiple *K* values (*K*=number of assumed clusters in the data) using the standard maximum *K* as the number of populations plus two: for *N. obliqua*, *K* = 1 to 22; *N. alpina*, *K* = 1 to 14; and *N. glauca*, *K* = 1 to 10. For each species, we performed 10 separate runs at each *K*. We used a burn-in period of 100,000 and 200,000 Markov chain Monte Carlo (MCMC) iterations for each run, obtaining posterior probabilities (*LnP[D]*) to detect the most likely *K*. We chose *K* at the highest *LnP[D]* and using Evanno's Δ*K* (Evanno et al. 2005), a criterion used to facilitate the selection of *K* when *LnP[D]* turns asymptotic. We employed Distruct 1.1 (Rosenberg 2004) to visualize and edit the Structure outputs.

After defining population clusters for each species using Structure, we employed locus-by-locus analysis of molecular variance (AMOVA) in Arlequin 3.11 (Excoffier et al. 2005) to partition within- and among-population genetic variation and, when applicable, genetic variation within and among clusters of populations. We conducted AMOVAs assuming SMM using *R*_ST_ (Slatkin 1995) and tested for significance with 1000 permutations. Additionally, we calculated *F*_ST_ values (Wright 1965) for comparison. From the *R*_ST_ values, we estimated the effective migration rate in number of individuals per generation (*N*_e_*m*) following the infinite island population model (Wright 1931). We also obtained pairwise *R*_ST_ matrices as measures of genetic differentiation among populations with 100 permutations to obtain significance using standard Bonferroni corrections on all pairwise differences (Rice 1989). We used Mantel tests (1000 permutations) to compare Slatkin linearized *R*_ST_ values and geographic distances in an isolation-by-distance (*IBD*) analysis, following the recommendation by Frantz et al. (2009) to measure *IBD* when interpreting Structure outputs.

Unrooted neighbor-joining (NJ) trees were constructed using pairwise *R*_ST_ matrices and the program Neighbor from Phylip 3.69 (Felsenstein 1989) to infer population similarities within species. We visualized the resulting trees with TreeView 1.6.6 (Page 1996). Before obtaining the pairwise *R*_ST_ matrices, we performed statistical tests for unequal contribution of loci in each species using AnimalFarm 1.0 (Landry et al. 2002), to exclude from the analysis any locus with too much contribution to the SMM-based distance coefficients (Landry et al. 2002).

#### Hybridization analysis

To determine the extent of hybridization among the three species, we looked for evidence of admixture using Structure 2.3.2 (Pritchard et al. 2000). We followed the same procedures described above, but in this case, we analyzed the populations of all three species together, totaling 640 individuals and 40 populations. After finding the optimal *K*, we evaluated the correspondence between the clusters and the morphological identity of the species to examine the admixture proportions found among species.

## Results

### Detection of scoring errors and linkage disequilibrium

Of all locus/population combinations, we identified 35% of the homozygote excesses to be due to null alleles in *Nothofagus obliqua*, 8% in *N. alpina*, and 9% in *N. glauca*. We corrected approximately half of the cases in Micro-Checker (van Oosterhout et al. 2004) and regarded the uncorrectable cases as missing data. Locus *NnBIO111* in *N. obliqua* had putative null alleles in all 20 populations, and most of them could not be corrected using Micro-Checker. Therefore, we excluded locus *NnBIO111* from all further analyses in this species (Table 3).

Combining *N. obliqua*, *N. alpina*, and *N. glauca*, we found 16 cases of significant linkage disequilibrium (LD, *α* = 0.05) over 487 possible combinations (3.3%), and none was consistent across populations. The most consistent event was the LD between *ncutas06* and *ncutas13*, but it was only significant in three of 40 populations, indicating that it was probably a relationship due to chance alone. All other cases of LD were significant in only one or two populations.

### Genetic diversity within populations

While we did not measure polymorphism in our study because it is usually close to 100% when using microsatellite loci selected specifically for their polymorphism, there was one monomorphic locus (*ncutas04*) in all populations of *N. obliqua* and *N. alpina*, but not in *N. glauca* (Table 3). In contrast, the other two measures of genetic diversity (i.e., *A* and *H*_E_) were always lower in *N. glauca* than in *N. alpina*, and lower in *N. alpina* than in *N. obliqua* (Table 4). Pairwise nonparametric among-species comparisons yielded highly significant results between *N. obliqua* and *N. glauca* for both parameters (*P* < 0.001), significant results between *N. obliqua* and *N. alpina* for *A* (*P* < 0.05), and between *N. alpina* and *N. glauca* for *H*_E_ (*P* < 0.05). None of the populations from any of the species had significant signs of recent genetic bottlenecks, with *P*-values always greater than 0.5 for the bottleneck analysis.

**Table 4:** Within-population diversity indices for *Nothofagus obliqua*, *N. alpina*, and *N. glauca*

#	Population	*N*	Loci	*A*	*H*_O_	*H*_E_	*F*_IS_	*ρ*_IS_
*N. obliqua*								
1	La Campana	15.8	4	6.5	0.493	0.779	0.376*	0.371
2	Loncha	14.8	5	7.0	0.681	0.714	0.044	0.072
3	Bellavista	15.2	5	6.2	0.589	0.688	0.147	0.090
4	Siete Tazas Alto	15.4	5	5.0	0.553	0.597	0.077	0.125
5	Bullileo Alto	14.4	5	5.4	0.579	0.620	0.070	−0.028
6	Ninhue	15.8	5	6.8	0.609	0.711	0.147	0.088
7	Recinto	15.6	5	6.8	0.632	0.689	0.086	0.042
8	Reserva Ñuble	16.0	4	7.0	0.609	0.683	0.111	−0.054
9	Santa Barbara	15.8	5	5.8	0.560	0.617	0.095	0.028
10	Ralco	14.6	5	4.8	0.438	0.534	0.192	0.043
11	Victoria	14.4	5	5.6	0.546	0.568	0.045	−0.092
12	Pichipillahuen	15.5	4	5.8	0.598	0.621	0.038	−0.042
13	Galletue	14.6	5	6.8	0.683	0.770	0.112	0.293
14	Cunco	15.6	5	7.4	0.659	0.680	0.031	0.396
15	Curarrehue	15.2	5	7.0	0.679	0.697	0.022	0.035
16	Cruces	15.0	5	7.0	0.655	0.685	0.046	0.189
17	Malalhue	15.8	5	6.8	0.573	0.631	0.094	0.254
18	Choshuenco	15.0	5	6.6	0.645	0.648	−0.003	0.294
19	Llancacura	15.2	5	6.2	0.614	0.659	0.070	0.188
20	Purranque	15.4	5	6.8	0.610	0.658	0.080	0.192
	Average	15.3	4.9	6.2	0.600	0.662	0.094	0.124
*N. alpina*								
21	Siete Tazas	15.8	6	3.8	0.485	0.419	−0.165	−0.187
22	Tregualemu	16.0	6	4.3	0.688	0.649	−0.062	−0.007
23	Bullileo Alto	15.8	6	4.7	0.494	0.541	0.088	0.072
24	Recinto	16.0	6	6.8	0.719	0.717	−0.003	−0.190
25	Nahuelbuta	15.8	6	7.3	0.746	0.761	0.018	0.016
26	Santa Barbara	15.8	6	5.3	0.666	0.555	−0.206	−0.260
27	Jauja	16.0	6	4.8	0.563	0.545	−0.033	−0.094
28	Pichipillahuen	16.0	6	5.7	0.625	0.572	−0.097	−0.524
29	Curarrehue	16.0	6	5.8	0.760	0.665	−0.148	−0.358
30	Releco	16.0	6	5.7	0.604	0.621	0.027	0.165
31	Llancacura	15.3	6	6.2	0.690	0.719	0.036	0.051
32	Hueyusca	14.5	6	5.2	0.396	0.635	0.382*	0.349
	Average	15.8	6.0	5.5	0.620	0.617	−0.014	−0.081
*N. glauca*								
33	Loncha	14.9	7	4.7	0.448	0.543	0.181	0.380
34	Alto Huelon	16.0	6	5.2	0.552	0.548	−0.007	0.123
35	Siete Tazas	15.0	7	4.0	0.394	0.497	0.215	0.296
36	Los Ruiles	15.9	7	4.1	0.460	0.480	0.043	−0.017
37	Tregualemu	14.8	6	4.2	0.476	0.522	0.094	0.042
38	Bullileo	15.9	7	4.3	0.448	0.455	0.018	0.149
39	Alico	15.1	7	5.7	0.441	0.535	0.184	0.131
40	Quilleco	15.7	7	4.1	0.436	0.431	−0.011	0.101
	Average	15.4	6.8	4.5	0.457	0.502	0.089	0.151

*N*, average sample size; loci, number of loci effectively analyzed; *A*, mean number of alleles per locus; *H*_*O*_, average observed heterozygosity; *H*_*E*_, average expected heterozygosity; *F*_*IS*_, inbreeding coefficient (Wright 1965); *ρ*_*IS*_, allele size-based inbreeding coefficient (Rousset 1996).

* Significant deviation from Hardy–Weinberg equilibrium (*α* = 0.05) after standard Bonferroni correction (Rice 1989).

Even though only populations La Campana (1) and Hueyusca (32) of *N. obliqua* and *N. alpina*, respectively, consistently showed significant deviations from HWE (*α* = 0.05) across all loci, there is a general tendency in *N. obliqua* and *N. glauca* to have positive inbreeding coefficients, *F*_IS_ and *ρ*_IS_, across populations, in clear contrast to *N. alpina* (Table 4).

In general, there was no significant correlation or consistent trend between diversity measures and latitude or elevation for the species. Only in comparisons between populations from the Coast and the Andes was there a general trend of higher levels of genetic variation in the Coast for all species, but this result was not significant.

We hypothesized that samples obtained from provenance trials would show inbreeding and lower levels of genetic diversity in comparison with samples from natural populations because in the trials, six of the16 sampled individuals were half-siblings. However, nonparametric Kruskal–Wallis tests did not show any significance or trends showing higher inbreeding or lower genetic diversity in samples obtained from the trials in either *N. obliqua* or *N. alpina* (all *P*-values > 0.5).

To avoid the confounding effect that source of plant material could have in comparing northern and southern groups of populations, we combined *N. obliqua* and *N. alpina* to contrast the levels of genetic diversity between these two latitudinal groups: north, including all populations from the Mediterranean forests north of the parallel 36°30′S, and south, including all others (Fig. 1, Table 2). *Nothofagus glauca* was not included in this analysis because seven of the eight populations are from the Mediterranean forests, and inclusion would have confounded the latitude and species effects. A general trend of less genetic diversity in the north than in the south was not statistically significant. Combining populations of *N. obliqua* and *N. alpina*, the tests comparing glacial refugia hypotheses were not significant and did not show any general trends. Populations south of 40°00′S were not lower in genetic diversity than northern populations, as the low-latitude refugia hypothesis predicts. The levels of genetic diversity in the southern populations (Table 4) agree with the multiple refugia hypothesis.

### Genetic structure inferred from Bayesian clustering

**Figure 2:**
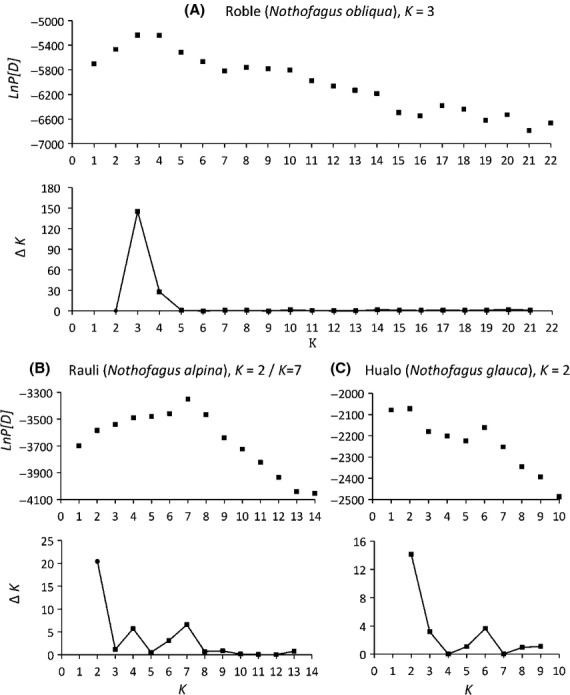
Log posterior probabilities (*LnP[D]*) and Δ*K* values (Evanno et al. 2005) against *K* (number of population clusters). We obtained all values using Structure 2.3.2 (Pritchard et al. 2000) for (A) 22 potential clusters in *Nothofagus obliqua*, (B) 14 in *N. alpina*, and (C) 10 in *N. glauca*. We chose the most likely *K* in each species at the highest *LnP[D]* and Δ*K* values. We identified three, seven, and two clusters in *N*. *obliqua*, *N. alpina*, and *N. glauca,* respectively.

The Bayesian analysis performed for each species separately yielded log posterior probabilities (*LnP[D]*) for each *K* (number of assumed clusters). In all three species, the number of optimal clusters was much lower than the number of sampled populations, indicating extensive gene flow, either historical or ongoing. The maximum *LnP[D]* was at *K* = 3 for *N. obliqua*, *K* = 7 for *N. alpina*, and *K* = 2 for *N. glauca* (Fig. 2). While all three *LnP[D]* curves had clear peaks and none of them turned asymptotic with the increase in *K*, we still plotted Evanno's Δ*K* (Evanno et al. 2005) to compare the estimates of *K* and obtain an idea of the signal strength at the optimal *K*. In *N. obliqua* (strong signal) and *N. glauca* (weak signal), Δ*K* agrees with the optimal *K* found by *LnP[D]*, but in *N. alpina* (weak signal), Δ*K* does not agree with the *K* found by *LnP[D]* and supports an optimal value of *K* = 2 instead of 7 (Fig. 2B).

**Figure 3:**
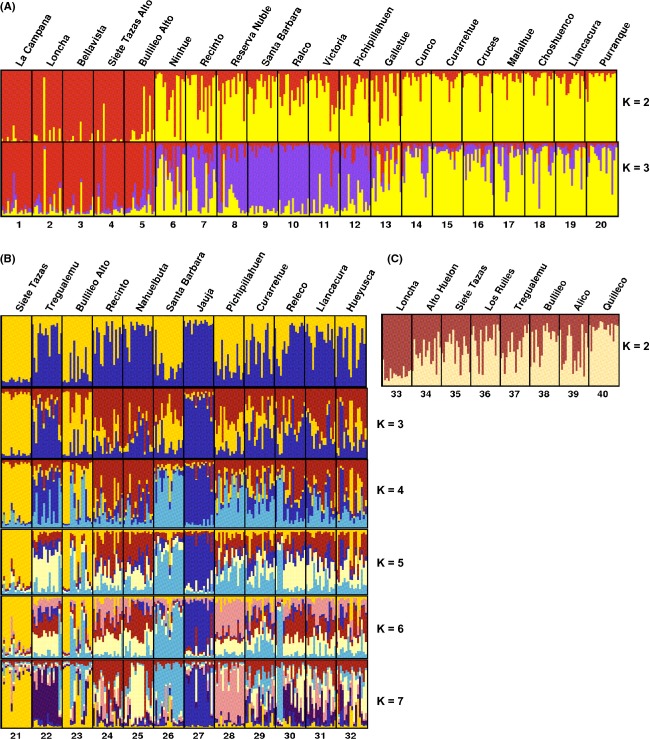
Results of Structure 2.3.2 analysis (Pritchard et al. 2000) showing from *K* = 2 to the most likely *K* in (A) *Nothofagus obliqua*, (B) *N. alpina*, and (C) *N. glauca*. Clusters of populations are represented by colors. Populations are defined by vertical lines and ordered north to south within each species. Within individuals, the proportion of each color indicates membership to the given cluster. We employed Distruct 1.1 (Rosenberg 2004) to visualize and edit the Structure outputs.

For *N. obliqua* (Fig. 3A) with *K* = 3, the northern populations (1–5), corresponding to the Mediterranean forests, are clearly differentiated from all other populations. Among the latter, a transition group (populations 7–12) is distinct from the southern populations (13–20), which correspond to the temperate rainforests. Only Ninhue (6), which belongs geographically to the northern group, clusters with populations from the south. Significant gene flow among groups is inferred from the levels of admixture seen in the Structure diagram (Fig. 3A).

For *N. alpina* (Fig. 3B) with *K* = 2 or *K* = 7, there is no geographic differentiation among populations. Moreover, the membership charts from *K* = 2 to *K* = 7 show a lack of identity in the populations. The only two populations that group together regardless of the number of clusters are Siete Tazas (21) and Bullileo Alto (23). Both populations are located in the Andes, in the northern tip of the distribution of *N. alpina*, separated by 100 km and relatively isolated from other populations (Fig. 1). Other populations that show a membership in a cluster greater than 50% are Tregualemu (22), Santa Barbara (26), Jauja (27), and Pichipillahuen (28).

For *N. glauca* (Fig. 3C) with *K* = 2, the northernmost population Loncha (33) and the southernmost population Quilleco (40) are clearly distinct and also very isolated from other populations of the species (Fig. 1). The remaining sampled populations (34, 35, 36, 37, 38, and 39) show admixture between the two clusters.

### Genetic structure inferred from AMOVA and pairwise genetic distances

The within-species AMOVAs indicated that the genetic variation within populations was always greater than 80%. The *R*_ST_ values were 0.11, 0.16, and 0.087 for *N. obliqua*, *N. alpina*, and *N. glauca*, respectively, representing low to moderate but statistically highly significant (*P* < 0.00001) levels of genetic differentiation among populations (Table 5). Using the *R*_ST_ values, we estimated the effective rates of migration (*N*_e_*m*) as 2.0, 1.3, and 2.6 individuals per generation, respectively. The *R*_ST_ values were similar to those obtained using the *F*_ST_ statistic, with *F*_ST_ = 0.12, 0.14, and 0.074 for *N. obliqua*, *N. alpina*, and *N. glauca*, respectively. In *N. obliqua*, Structure also found that the species could be divided into three spatially defined and homogeneous groups of populations. As a result, we ran an AMOVA including that extra group level. In this analysis, the variation among groups (*R*_CT_) was 0.114 (*P* < 0.00001) and the variation among populations within groups (*R*_SC_) was only 0.037 (*P* < 0.00001), also indicating homogeneity within groups with substantially more intragroup (*N*_e_*m* = 6.5) than intergroup (*N*_e_*m* = 1.9) migration. Consequently, the total variation among populations (*R*_ST_) was 0.146 (*P* < 0.00001), higher than the original values for *N. obliqua* (*R*_ST_ = 0.11), because we excluded Ninhue (6), which did not have a well-defined membership to any group.

**Table 5:** Global analysis of molecular variance (AMOVA) showing the partition of genetic variation among and within populations for *Nothofagus obliqua*, *N. alpina*, and *N. glauca*

Source of variation	df^1^	Sum of squares	Variance components	Percentage of variation	*R*_ST_^2^
*N. obliqua*					
Among populations	19	2449.8	3.274	11.0	0.110
Within populations	606	16,108.3	26.519	89.0	
Total	625	18,558.1	29.793		
*N. alpina*					
Among populations	11	3009.8	7.430	16.0	0.160
Within populations	366	14,290.3	38.910	84.0	
Total	377	17,300.1	46.340		
*N. glauca*					
Among populations	7	834.4	2.909	8.7	0.087
Within populations	241	7264.9	30.367	91.3	
Total	248	8099.3	33.276		

Results are a weighted average over usable loci. We performed the analyses under the stepwise mutation model (SMM) using *R*_ST_-like sum of squared size differences with 1000 permutations.

1 Average degrees of freedom across loci.

2 All *R*_ST_ are highly significant (*P* < 0.00001).

**Figure 4:**
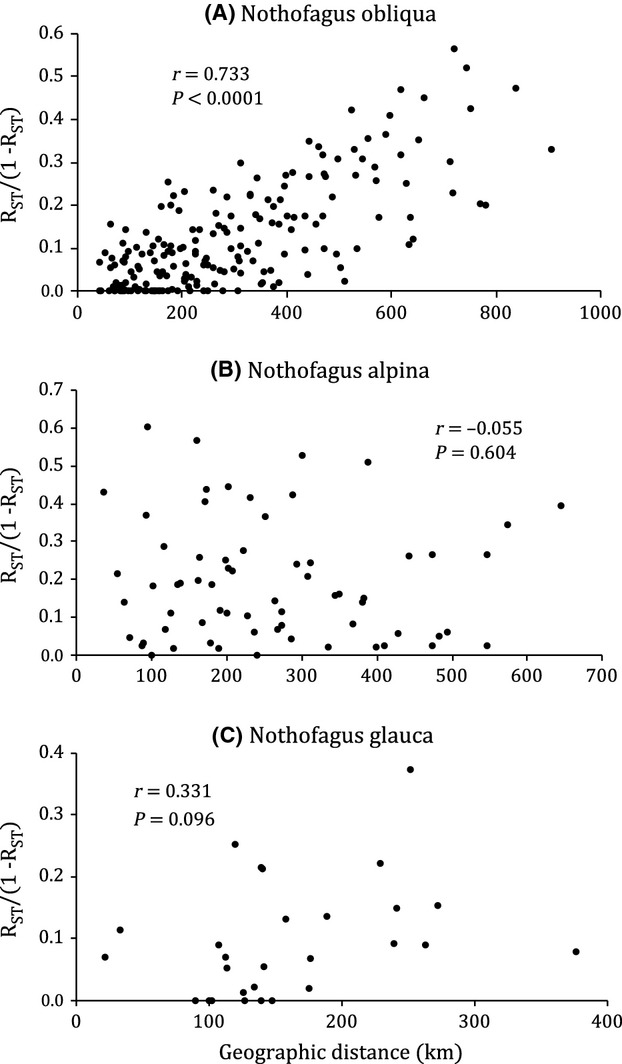
Correlations between pairwise Slatkin linearized *R*_ST_ (Slatkin 1995) and pairwise geographic distances (Mantel tests) to evaluate isolation by distance (*IBD*) in (A) *Nothofagus obliqua*, (B) *N. alpina*, and (C) *N. glauca*. We obtained Pearson correlation coefficients (*r*) and *P*-values (*P*) for each test.

The Mantel tests we conducted between pairwise Slatkin linearized *R*_ST_ (Slatkin 1995) and geographic distance matrices to test for *IBD* showed a strong and highly significant relationship between genetic and geographic distances in *N. obliqua* (*r* = 0.73; *P* =< 0.0001; Fig. 4A), no relationship in *N. alpina* (*r* = −0.06, *P* = 0.604; Fig. 4B), and a positive but weak relationship in *N. glauca* (*r* = 0.33, *P* = 0.096; Fig. 4C).

**Figure 5:**
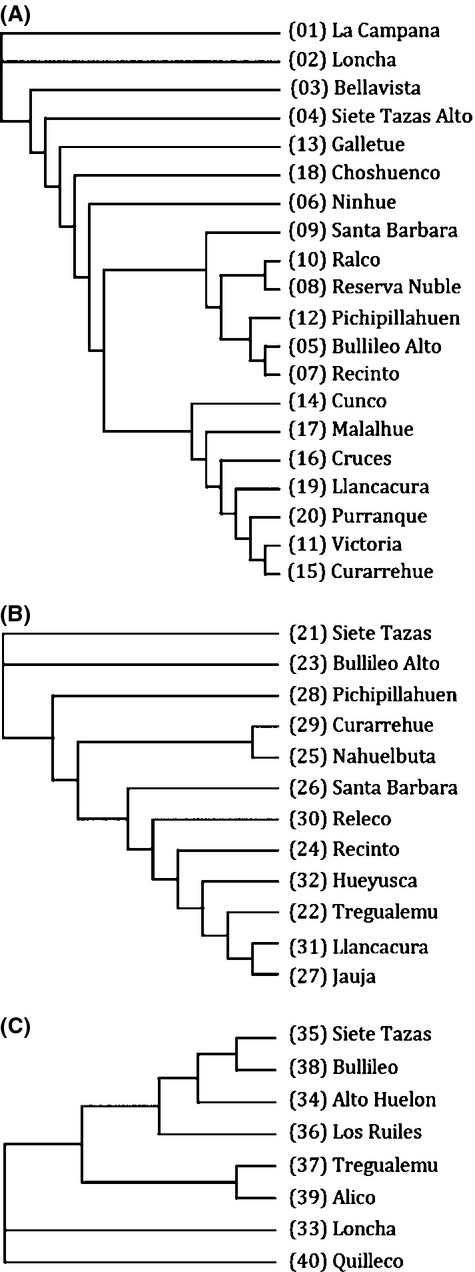
Unrooted neighbor-joining trees obtained in Phylip 3.69 (Felsenstein 1989) using *R*_ST_ values for all pairs of sampled populations in (A) *Nothofagus obliqua*, (B) *N. alpina*, and (C) *N. glauca*.

After confirming that there were not significant inequalities in the contribution of loci to the SMM-based distance coefficients, we obtained unrooted NJ trees from the pairwise *R*_ST_ matrices for each species (Fig. 5). In *N. obliqua* (Fig. 5A), there are two defined branches in the NJ tree: one composed only of populations from the temperate rainforests in the south, including Llancacura (19) and Purranque (20), and another composed of populations from the central and transitional part of the distribution, including Reserva Ñuble (8) and Ralco (10). None of the populations from the Mediterranean forest in the north from La Campana (1) to Ninhue (6), except Bullileo Alto (5), clustered with any other population. Interestingly, two populations from the south, Galletue (13) and Choshuenco (18), also failed to cluster with other populations.

For *N. alpina* (Fig. 5B), there are no clear clusters in the tree, and the genetic similarities among populations do not agree in general with their geographic distributions. However, similar to *N. obliqua*, the two populations from the Andes in the northern portion of the distribution of the species appear genetically isolated from the rest of the populations and from each other. *Nothofagus glauca* (Fig. 5C) exhibits greater association between geographic and genetic distances than the other two species, with three main branches in the NJ tree. The extreme populations Loncha (33) in the north and Quilleco (40) in the south are genetically separated from each other and from the rest and geographically isolated. The rest of the populations fall in one cluster with variable levels of isolation.

### Hybridization analysis

By running Structure and including all populations from the three species together, we obtained the optimal number of clusters, *K* = 3, with a high correspondence (>90%) with the morphological identity of the species for all *N. alpina* and *N. glauca* populations, and for populations (6) to (20) in *N. obliqua*. Northern populations (1) to (5) in *N. obliqua* had higher admixture, but their identity was still greater than 50% (data not shown). Under this scenario, in *N. obliqua* there was an average admixture proportion of 10.3% coming from *N. alpina* and 1.9% coming from *N. glauca*, far higher than the admixture going to *N. alpina* or *N. glauca* (Table 6).

**Table 6:** Membership proportions averaged over all populations of *Nothofagus obliqua*, *N. alpina*, and *N. glauca* individuals in the inferred clusters obtained using Structure.

	Clusters (for *K* = 3)			Clusters (for *K* = 4)				
		
Species	1	2	3	1^1^	2^1^	1 + 2	3	4
*N. obliqua*	0.878	0.103	0.019	0.311	0.641	0.952	0.034	0.014
*N. alpina*	0.022	0.972	0.006	0.037	0.013	0.050	0.944	0.006
*N. glauca*	0.016	0.009	0.975	0.011	0.015	0.026	0.007	0.967

1 Clusters 1 and 2 represent northern and southern *N. obliqua* populations, respectively.

It is clear, however, that the admixture in *N. obliqua* is not distributed evenly among all populations; most of it is in the northern populations. Due to this uneven distribution in admixture proportions, we also inspected data using *K* = 4, which we knew placed these northern populations in a different cluster, allowing us to have a separate estimation of admixture. Most of the interspecies admixture in *N. obliqua* disappeared in this analysis, reducing the overall admixture proportion in *N. obliqua* to 3.4% coming from *N. alpina* and 1.4% coming from *N. glauca*, with only minor changes to the values going to *N. alpina* and *N. glauca* (Table 6). In addition, the distribution of the admixture in *N. obliqua* is more even, with identities always greater than 90%, except in La Campana (1) and Loncha (2) with 86% each.

## Discussion

### Genetic variation within populations at nuclear microsatellite loci

The high levels of nuclear microsatellite genetic variation in these species of *Nothofagus* are consistent with results for other related, large-sized, long-lived, outcrossing, wind-pollinated trees (Table 1). For nuclear microsatellite loci, values for genetic diversity (*H*_E_ = 0.452–0.496) and number of alleles per locus (*A* = 3.7–4.4) for *Nothofagus* in Argentina (Milleron et al. 2008; Azpilicueta et al. 2013; Mathiasen and Premoli 2013) and for species of the related genera *Fagus* and *Quercus* (0.597–0.864 for *H*_E_, 4.9–14.9 for *A*) from the Northern Hemisphere are similar to those found in our study (*H*_E_ = 0.50–0.66, *A* = 4.5–6.2). However, our values are higher than those reported for *Nothofagus* in Argentina and fall in the lower end of the ranges for *Fagus* and *Quercus*. We attribute the latter to the span of the geographical distributions of the species in our study, which are more narrowly distributed than those species from the Northern Hemisphere.

*Nothofagus alpina* from Chile has lower RAPD diversity than other long-lived forest species, a result attributed to intense exploitation and substitution during the last century (Carrasco et al. 2009). Taylor et al. (2005) found values of ISSR diversity in Australian *N. moorei* similar to those reported by Carrasco et al. (2009). However, *N. moorei* is an isolated species with a very narrow distribution. Interestingly, several reports studying allozyme variation in *N. alpina* and *N. obliqua* found levels of variation similar to (Marchelli and Gallo 2001, 2004; Azpilicueta and Gallo 2009) and even higher than (Pineda 2000; Carrasco and Eaton 2002) those for species of other related genera, including *Castanea*, *Quercus*, and *Fagus*.

Our results indicate that despite a century of exploitation and substitution, the dramatically reduced fragments of forest examined here probably still contain an important fraction of their neutral genetic variability, agreeing with the findings of Craft and Ashley (2007) for *Quercus macrocarpa* in Illinois, USA, but not with what Carrasco et al. (2009) hypothesize for *N. alpina*, or Torres-Diaz et al. (2007) for *N. alessandrii* in Chile. Also, based on the relatively high levels of variation and the lack of evidence for recent bottlenecks found in our study and in Carrasco and Eaton (2002), populations from these three species appear to have overcome any genetic impact of the severe climatic changes that occurred during the last 20,000 years. Hamrick (2004) explains the resilience of forest trees to climatic change and habitat fragmentation, indicating that characteristics such as individual longevity, high neutral within-population genetic variation, and extensive pollen flow may make them especially resistant to the loss of genetic diversity and further extinction in changing environments.

### Differences in within-species genetic variation

*Nothofagus obliqua*, *N. alpina*, and *N. glauca* have relatively high levels of genetic variation, as shown by values of *A* and *H*_E_. Yet, there are statistically significant differences between these species, and those differences correspond to their geographic ranges. *Nothofagus glauca*, with a narrow distribution, has the lowest values of genetic diversity in both parameters, followed by *N. alpina* with a regional distribution. Finally, *N. obliqua*, also with a regional distribution but somewhat larger than that of *N. alpina*, is the most genetically diverse species. These findings agree with Hamrick and Godt (1996), who found that geographic range is one of the predictors of genetic diversity. A wider distribution may help to generate and maintain genetic variability, or conversely, high genetic variability may improve the chances of colonizing heterogeneous environments.

Although it may be misleading to compare this range-influenced trend of genetic variation obtained for the Chilean species of subgenus *Lophozonia* with other genera (Gitzendanner and Soltis 2000), the trends in the levels of genetic diversity (*H*_E_) in relation to geographic range are consistent with general expectations. Using the studies with nuclear microsatellite markers for *Fagus* and *Quercus* listed in Table 1, we find larger genetic diversity values (*H*_E_ = 0.68–0.87) for widespread species such as *F. sylvatica* and intermediate values (*H*_E_ = 0.60–0.66) for regional species such as *Q. garryana*, similar to values for *N. alpina* (*H*_E_ = 0.62) and *N. obliqua* (*H*_E_ = 0.66). Accordingly, narrowly distributed *N. glauca* has lower diversity (*H*_E_ = 0.50).

### Outcrossing and inbreeding

Species of *Nothofagus* have a largely outcrossing breeding system (Riveros et al. 1995; Gallo et al. 1997; Ipinza and Espejo 2000). However, the occurrence of at least some inbreeding in natural populations of *Nothofagus* (e.g., Premoli 1997; Pineda 2000; Carrasco and Eaton 2002) and also in member of the closely related Fagaceae (e.g., Muir et al. 2004; Lee et al. 2006; Buiteveld et al. 2007; Marsico et al. 2009) is the rule. This inbreeding is probably because forest stands are composed of groups of related trees that allow breeding between cousins, parents and offspring, half-sibs, and even full-sibs. This may explain why samples obtained from provenance trials (some of them half-sibs) had neither lower genetic variation nor higher inbreeding coefficients than the natural populations.

In our study, after correcting for null alleles, we found only two statistically significant departures from Hardy–Weinberg equilibrium (HWE): La Campana (1) in *N. obliqua* and Hueyusca (32) in *N. alpina*. However, despite the lack of significant inbreeding for most of the populations, there is a tendency for inbreeding coefficients (i.e., *F*_IS_ and *ρ*_IS_) to be greater than zero in *N. obliqua* and *N. glauca* (Table 4). This consistency suggests that with a larger sample size, higher, and perhaps statistically significant, levels of inbreeding might be detected in these two species. Also, it is important to keep in mind that the algorithm used to detect and correct null alleles could have masked some true inbreeding. In *N. alpina*, there is no trend among populations; thus, with the exception of Hueyusca (32), the *F*_IS_ and *ρ*_IS_ values are probably due to chance alone. The general correspondence of populations of *N. alpina* with HWE could mean that this species has more efficient ways of avoiding inbreeding such as larger effective population sizes or its regeneration strategy, but so far all the evidence in the literature states the opposite (Pineda 2000; Carrasco and Eaton 2002; Marchelli and Gallo 2004).

With regard to the inbred populations, it is reasonable that La Campana (1) at the northern limit of *N. obliqua* and Hueyusca (32) at the southern limit of *N. alpina* (Fig. 1) showed evidence of inbreeding because of their isolation and relatively small population sizes. However, it is not apparent why only those populations would be inbred and not other similarly small populations such as Loncha (2) and Bellavista (3) in the north or Llancacura (31) in the south. Further research will be necessary to examine these issues.

### Structure and isolation by distance (IBD)

According to simulations by Frantz et al. (2009), it appears that strong *IBD* would have an effect on the clustering algorithm in Structure, mainly by making the posterior probability of the data become asymptotic, thus overestimating the number of “real” clusters. Fortunately, even though we have strong evidence of *IBD* in *N. obliqua* and some evidence in *N. glauca*, the posterior probabilities in our Structure analyses were not asymptotic and had a peak that was also coincident with the optimal number of clusters given by Δ*K*. In addition, Frantz et al. (2009) claim that clusters obtained by Structure in these conditions of strong *IBD* have too much overlap. In our case, particularly with *N. glauca*, this is true. However, we believe that overlap is precisely a product of what is really going on in the distribution of variation, and we prefer not to consider possible clusters where the variation may actually be clinal.

#### Structure in *Nothofagus obliqua*

**Figure 6:**
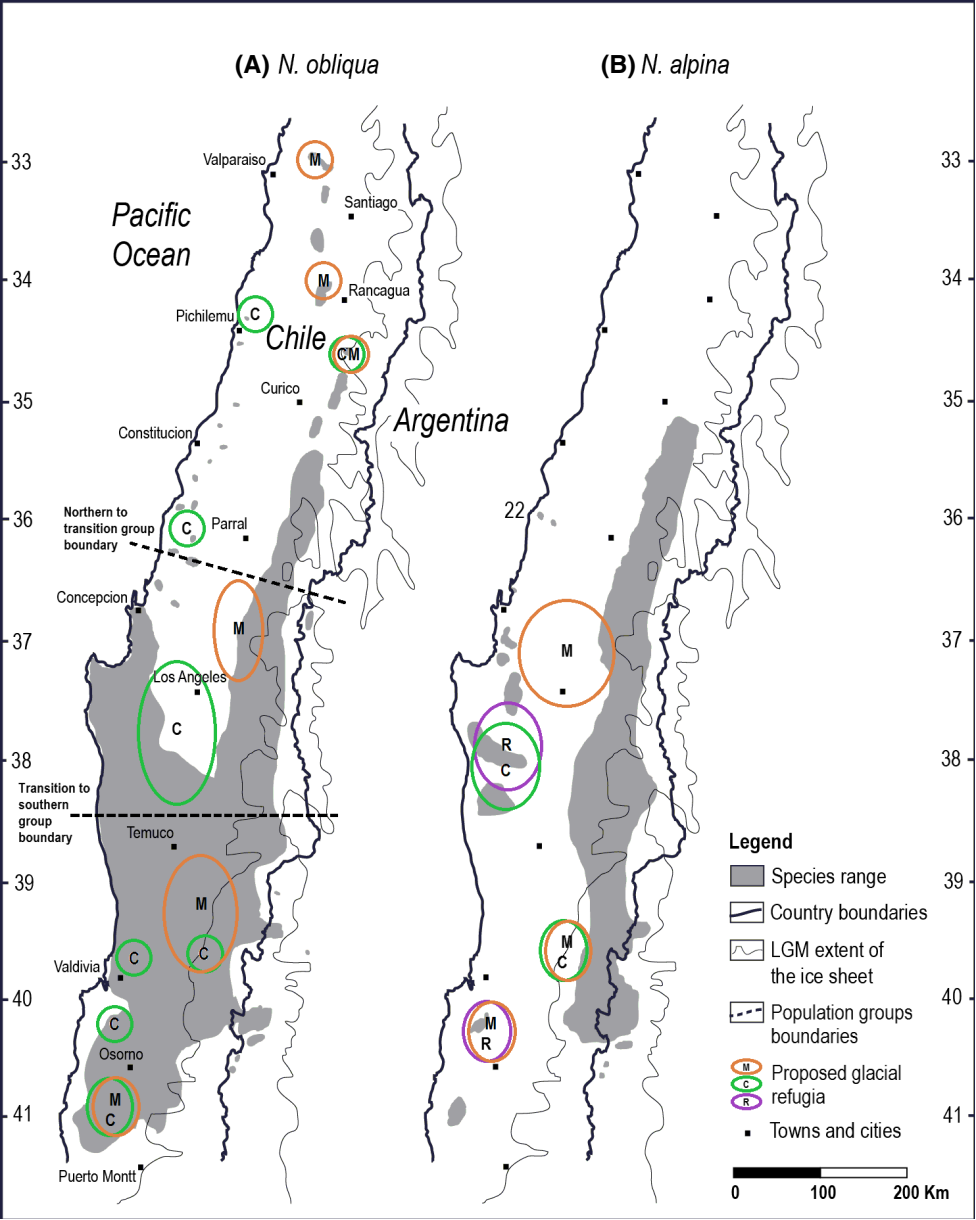
Proposed glacial refugia for (A) *N. obliqua* and (B) *N. alpina* based on our microsatellite data (M, orange). Proposed glacial refugia based on chloroplast DNA (C, green) (Marchelli and Gallo 2006; Azpilicueta et al. 2009), and RAPDs (R, purple) (Carrasco et al. 2009) are shown for comparison. For *N. obliqua*, the boundaries between population groups defined by Bayesian clustering in Structure 2.3.2 are also presented. Last glacial maximum extent of the ice sheet obtained from Hollin and Schilling (1981).

The percentage of among-population neutral variation found for *N. obliqua* (11%) is moderate in comparison with values for *N. alpina* and *N. glauca* in our study, and with other Nothofagaceae and Fagaceae (Table 1). This indicates that gene flow (*N*_e_*m* = 2.0) maintains the genetic homogeneity of the species quite well. Additionally, *IBD* plays an important role (Fig. 4A), suggesting that a good part of the interpopulational variability is clinal and not due to abrupt differences between populations. We did, however, find a relatively clear subdivision into three homogeneous groups of populations (Figs. 3A, 6A). There is gene flow among these groups (*N*_e_*m* = 1.9), but it is more restricted than the gene flow among populations within groups (*N*_e_*m* = 6.5). These groups are distributed latitudinally, each with members from very different environments (Table 2), indicating extensive genetic interchange among elevations and locations that apparently do not challenge local adaptation.

The subdivision into three groups (Fig. 6A) also agrees in general with the division into two forest types and an ecotonal zone proposed by Donoso (1982) and Veblen and Schlegel (1982), suggesting that around the southern limit of the Mediterranean forests at latitude 36°30′S, there is a geographic barrier – probably the Ñuble River valley, slowing colonization and pollen flow and creating a divide between the northern and transitional groups. The divide between the transitional and southern groups at approximately latitude 38°30′S does not match any apparent geographic barrier to gene flow, but it coincides surprisingly well with a major change in climate passing from a dry-summer subtropical climate (*Csb*) to an oceanic climate (*Cfb*) based on the Köppen–Geiger classification (Fuenzalida 1965). The NJ tree (Fig. 5A) agrees fairly well with the two splits, but suggests that populations in the northern group have a higher level of genetic isolation from each other than the populations in the other groups, consistent with the geographic isolation of these populations.

The organization of among-population genetic variability in *N. obliqua* follows a clinal pattern of variation similar to that described by Donoso (1979a), but with two recognizable “jumps” in the cline, possibly due to current features in the landscape as stated above, but also possibly due to past landscape features as suggested by Premoli et al. (2012) for other *Nothofagus* species. The study of *N. obliqua* by Azpilicueta et al. (2009) using chloroplast DNA (cpDNA) haplotypes recognized a different pattern of variation, showing that populations in the Coastal Range are very distinct from each other and also different from those from the Central Valley or the Andes, indicating that the genetic homogeneity of our three latitudinal groups is due mainly to pollen flow rather than recent range expansion. However, the pattern of variation in the Central Valley and in the Andes in Chile is similar to that found in our study, following a north to south line, and with a divide at approximately the same latitude as our south-transition boundary (38°30′S). Azpilicueta et al. (2009) did not find a divide at the Ñuble River valley at latitude 36°30′S, but did at 35°00′S, with the isolated populations on the highlands of the north of the distribution forming their own cluster.

#### Structure in *Nothofagus alpina*

Among-population neutral variation in *N. alpina* was the highest in our study (16%, *N*_e_*m* = 1.3), and it is among the highest values reported for *Nothofagus* and the closely related Fagaceae (Table 1). This value might be larger than in *N. obliqua* because the *N. alpina* coastal populations are more isolated from each other and from the Andean populations. Furthermore, the level of among-population variation combined with the absence of *IBD* in this species (Fig. 4B) indicates the presence of selective barriers to gene flow, and the absence of apparent geographical groups (Fig. 3B) supports this idea. The clinal pattern found in *N. obliqua* is not repeated in *N. alpina*. Figure 5B also shows a lack of spatial structure, with high genetic similarities between very distant populations such as Nahuelbuta (25) and Curarrehue (29) or between Tregualemu (22), Jauja (27), and Llancacura (31).

Our findings do not agree in general with the genetic clusters obtained for the Chilean populations of *N. alpina* using allozymes (Pineda 2000; Carrasco and Eaton 2002) and RAPDs (Carrasco et al. 2009). These studies show an overall correspondence between genetic and spatial patterns of variation, although in all of them there are cases of populations clustering with geographically distant groups. Marchelli and Gallo (2006) used cpDNA haplotypes to describe the patterns of variation in *N. alpina*, finding no breaks among populations on the Chilean side of the Andes, and isolated haplotypes in two populations sampled in the Coastal Range, agreeing with a spatially undefined structure.

#### Structure in *Nothofagus glauca*

In the narrowly distributed *N. glauca*, among-population neutral variation (8.7%) is lower than those values for *N. obliqua* and *N. alpina* but still moderate in comparison with other *Nothofagus* and Fagaceae (Table 1), indicating moderate to high levels of gene flow among populations (*N*_e_*m* = 2.6). There is a weak tendency for the populations to be isolated by distance (Fig. 4C), but the two populations driving this tendency – the northernmost population Loncha (33) and the southernmost population Quilleco (40) – are also very isolated from the core range of *N. glauca* (Fig. 1). Within the six populations of this core, there is no *IBD,* and there is apparent east/west gene flow, as suggested by the genetic similarity between Coastal Tregualemu (37) and Andean Alico (39) and the genetic dissimilarity between Bullileo (38) and Alico (39), two Andean populations geographically close but on different sides of a group of mountains (Figs. 1, 5C).

The results of the Structure analysis also support extensive gene flow among populations and the greater isolation of Loncha (33) and Quilleco (40), with no apparent geographical groups (Fig. 3C). Unfortunately, there is virtually nothing in the literature about the patterns of genetic or morphological variation in this species to support or dispute our results.

### Contribution to genetic variation from hybridization

A potential source of genetic variation and structure in the studied populations is hybridization within the subgenus. Natural F_1_ hybrids have been found in specific environmental conditions, that is, *N. alpina* × *N. obliqua* (Donoso et al. 1990; Gallo et al. 1997; Marchelli and Gallo 2001), *N. obliqua* x *N. glauca* (= *N. leonii* Espinosa) (Donoso 1979b), and populations with different levels of introgression mainly toward *N. obliqua*. The level and sources of admixture we obtained in Structure (Table 6) agree with the hybridization and introgression patterns proposed previously within the subgenus, suggesting that they reflect real events involving the sampled populations. Given that we sampled only populations with an unequivocal morphological identity for each species, evidence of admixture indicates that some hybridization and introgression must have occurred in the past. However, the signs of admixture between *N. alpina* and *N. glauca* are probably artifacts because they appear in low frequency (less than 1%), many cases occur in populations that never were in sympatry, and there are no reports of hybridization between these two species.

We examined two outcomes from the Structure analysis to interpret admixture among the three species. When using the optimal number of clusters (*K* = 3), with each species belonging to its own cluster, we found high levels of gene flow going from *N. alpina* toward *N. obliqua* in the highlands of the northern part of the distribution in the Mediterranean forests between 33°00′S and 36°30′S. This result was very surprising because currently in this area the species do not seem to interbreed and the populations with more admixture, that is, La Campana (1), Loncha (2), and Bellavista (3), are isolated from any *N. alpina* populations. Interestingly, according to Vazquez and Rodriguez (1999), the *N. obliqua* populations from this area, which they believe should be regarded as *N. macrocarpa*, are morphologically more similar to *N. alpina* than to *N. obliqua* from the south. If these results are verified, they will be strong evidence of ancient hybridization between *N. obliqua* and *N. alpina* with marked introgression toward *N. obliqua* that probably occurred around 10,000 years ago in the glacial refugia located in the valleys between 33°00′S and 36°00′S (Heusser 1990). A similar case of ancient hybridization is suggested by Azpilicueta and Gallo (2009) for *N. obliqua* in Argentina, where long-time isolated populations of this species show high frequencies of alleles specific to *N. alpina*. Further, these two species share several chloroplast DNA haplotypes, indicating past capture events through interbreeding (Azpilicueta et al. 2009). Thus, past hybridization between *N. obliqua* and *N. alpina* may have been a major force in shaping the patterns of genetic structure in *N. obliqua*.

We also examined the suboptimal *K* = 4, at which Structure splits the *N. obliqua* populations precisely into one cluster composed of the northern populations and the other cluster containing the rest. This shows that admixture coming from *N. alpina* to *N. obliqua* is three times higher in the northern populations than in the rest, supporting the ancient hybridization/introgression hypothesis described above (Table 6).

### Natural history and the glacial refugia hypotheses

The effects of the last glacial maximum (LGM) on the distribution patterns and genetic variation of *N. obliqua* and *N. alpina* have been revealed by palynological and cpDNA studies. Pollen profiles obtained at different latitudes and elevations in Chile support the low-latitude glacial refugia hypothesis localizing refugia in low elevations north of 40°00′S and agree with the general tendency seen in the Northern Hemisphere of lower-latitude refugia (Soltis et al. 1997, 2006; Hewitt 2000). On the other hand, the distributions of haplotypes obtained through cpDNA analysis in both species coincide with the multiple glacial refugia hypothesis suggested for virtually all tree species in southern South America (Marchelli et al. 1998; Allnutt et al. 1999; Premoli et al. 2000, 2003; Bekessy et al. 2002; Marchelli and Gallo 2004, 2006; Nunez-Avila and Armesto 2006; Azpilicueta et al. 2009; Carrasco et al. 2009; Pastorino et al. 2009; Mathiasen and Premoli 2010). The multiple refugia hypothesis, including high-latitude refugia, is also postulated as an alternative hypothesis for some species in North America (Soltis et al. 1997, 2006) and proposed by Magri et al. (2006) in Europe for *Fagus sylvatica*, a species that was formerly described as an example of the low-latitude hypothesis (Demesure et al. 1996).

Our results agree with the multiple refugia hypothesis and, therefore, with the existence of several centers of genetic diversity across the entire geographical ranges of these species. Also, the lack of signs of bottlenecks or repeated founder events in our microsatellite data indicates that these refugia harbored considerable variation and that pollen flow has been an important agent in keeping populations genetically diverse and genetically connected from the time of the first recolonization events after the LGM.

Not all refugia contributed equally to the recolonization and later migrations. The cpDNA evidence for *Nothofagus obliqua* (Azpilicueta et al. 2009) and *N. alpina* (Marchelli and Gallo 2006), which traces seed movements, shows that most refugia in the Coastal Range and north of 35°00′S do not seem to have participated in the postglacial colonization and remained confined within their areas of origin. Our results using nuclear microsatellite data, which trace pollen movements, indicate that genes did move from the Andes to the Coastal Range refugia and vice versa after colonization, generating great among-population admixture in *N. obliqua* and somewhat restricted admixture in *N. alpina*.

Azpilicueta et al. (2009) showed that the Chilean populations of *N. obliqua* had two relatively widespread haplotypes currently present in the Central Valley and the Andes from 35°00′ to 41°00′S. They seem to have been generated in two glacial refugia: one in the valleys or Andean western piedmont around 39°30′S, and the other either in the Central Valley near the Nahuelbuta mountains (37°00′ to 38°30′S), in agreement with palynological records (Villagran 1991), or further north in the Mediterranean forests close to the population Bellavista (3) at 34°45′S. Our microsatellite analysis matches the division between populations south and north of 38°30′S, but shows that, based on the values of *A* (Widmer and Lexer 2001), populations immediately north of 38°30′S are less genetically diverse than those just to the south, suggesting the existence of one or more glacial refugia in the area just south of 38°30′S (Fig. 6A). Further north, our data indicate a divide at latitude 36°30′S that does not seem to match the genetic diversity patterns obtained by Azpilicueta et al. (2009). Thus, according to our measures of *A* (Table 4), a more probable location of a glacial refugium in the Andean piedmont would be between 36°20′ and 37°20′S, just south of the Mediterranean forests and north of the Nahuelbuta mountains. Also, according to our microsatellite data, the currently isolated populations north of 36°30′S in the Mediterranean forests are genetically homogeneous and seem to have originated from different glacial refugia located in the lowlands and piedmonts between 33°00′ and 35°00′S where current populations have higher *A* values. Finally, the population Purranque (20) in the Coast Range near 41°00′S seems to also be a glacial refugium for *N. obliqua* that, like most of the Coastal Range refugia, did not participate in postglacial colonization (Azpilicueta et al. 2009) but did participate in subsequent pollen flows.

In *N. alpina*, Marchelli and Gallo (2006) did not find clear cpDNA haplotypic divides in Chilean populations, and their data suggest a northward postglacial colonization of the Andes from a single southern refugium located around 39°45′S in the Andes. Our results partially support this colonization trend with a slight increase in diversity to the south (Table 4) and lack of spatially explicit groups of populations (Fig. 3). However, there is a marked increase in genetic diversity in populations Recinto (24) and Nahuelbuta (25), in the same area where we proposed the refugium for *N. obliqua* and Carrasco et al. (2009) proposed a refugium for *N. alpina*. Thus, this area becomes a plausible refugium for this species as well (Fig. 6B). Additionally, Carrasco et al. (2009) proposed a southern refugium located in the Coastal Range south of 40°00′S, which could have also contributed with seeds and pollen to the northward postglacial colonization of the Andes. Our data also agree with these findings, as population Llancacura (31) at 40°17′S presents the highest *A* value for *N. alpina* in the south (Table 4).

The population genetics of *N. glauca* (which mostly occurs in intermediate elevations in both mountain ranges) was probably only mildly influenced by the climatic changes since the last glaciation. Our data indicate that the limited genetic structure of this species is due to two very isolated populations (Fig. 1): the northernmost population Loncha (33), from the highlands of the Coastal Range, and the southernmost population Quilleco (40), from 100 km south of the main geographical range of the species (Le-Quesne and Sandoval 2001). The large and heavy *N. glauca* seeds make it difficult to believe that Quilleco (40) is the result of a 100-km dispersal event. More likely, there were small *N. glauca* stands in the Andean foothills and Central Valley connecting all the populations of the species during the LGM about 20,000 years ago when moist conditions at low elevations were ideal for maintaining *Nothofagus* forest. Drier conditions could have caused most of the populations to gradually disappear in the area, except Quilleco (40). Moreover, this population has been isolated from other putative *N. glauca* populations in the area by the great alluvial cone of Lake Laja deposited in the Laja valley about 10,000 years ago (Thiele et al. 1998).

## Conclusion

Our results indicate that the levels of neutral genetic variation exhibited by *N. obliqua*, *N. alpina*, and *N. glauca* are high and correspond to the values of other tree species with similar natural histories and ranges of distribution. There is limited, but significant, genetic structure explained by isolation by distance within population continua, geographic isolation of certain populations, and some hypothesized geographic barriers to gene flow. Also, the tendency of these species to form stands with different levels of kinship did not result in significant inbreeding. On the other hand, the evidence of ancient and current hybridization as an important contributor to the intraspecific genetic diversity within the subgenus indicates that it may be a major source of variability and differentiation for the northern populations of *N. obliqua*. Finally, the patterns of genetic variation that we observed agree with the multiple refugia hypothesis for *N. obliqua* and *N. alpina* and suggest several centers of genetic diversity across the areas of distribution for all three species.

## Data Accessibility

Microsatellite data available from the Dryad Digital Repository: http://doi.org/10.5061/dryad.h3d26 (note: we show sample locations in Table 2 in this paper representing the general area where each population was sampled from. We do not have the specific location of individual tree samples within populations).
